# Gut Microbial Dynamics during Conventionalization of Germfree Chicken

**DOI:** 10.1128/mSphere.00035-19

**Published:** 2019-03-27

**Authors:** Milton Thomas, Supapit Wongkuna, Sudeep Ghimire, Roshan Kumar, Linto Antony, Kinchel C. Doerner, Aaron Singery, Eric Nelson, Tofuko Woyengo, Surang Chankhamhaengdecha, Tavan Janvilisri, Joy Scaria

**Affiliations:** a Department of Veterinary and Biomedical Sciences, South Dakota State University, Brookings, South Dakota, USA; b South Dakota Center for Biologics Research and Commercialization, Brookings, South Dakota, USA; c Department of Biochemistry, Faculty of Science, Mahidol University, Bangkok, Thailand; d Department of Biology and Microbiology, South Dakota State University, Brookings, South Dakota, USA; e Department of Animal Science, South Dakota State University, Brookings, South Dakota, USA; f Department of Biology, Faculty of Science, Mahidol University, Bangkok, Thailand; University of California, Davis

**Keywords:** *Salmonella*, competetive exclusion, feral chicken, gnotobiotic, metagenome, microbiota

## Abstract

A gnotobiotic Gallus gallus (chicken) model was developed to study the dynamics of intestinal microflora from hatching to 18 days of age employing metagenomics. Intestinal samples were collected from a local population of feral chickens and administered orally to germfree 3-day-old chicks. Animals were euthanized on days 9 and 18 postinoculation, and intestinal samples were collected and subjected to metagenomic analysis. On day 18, the five most prevalent phyla were *Bacteroidetes* (43.03 ± 3.19%), *Firmicutes* (38.51 ± 2.67%), *Actinobacteria* (6.77 ± 0.7%), *Proteobacteria* (6.38 ± 0.7%), and *Spirochaetes* (2.71 ± 0.55%). Principal-coordinate analysis showed that the day 18 variables clustered more closely than the day 9 variables, suggesting that the microbial communities had changed temporally. The Morista-Horn index values ranged from 0.7 to 1, indicating that the communities in the inoculum and in the day 9 and day 18 samples were more similar than dissimilar. The predicted functional profiles of the microbiomes of the inoculum and the day 9 and day 18 samples were also similar (values of 0.98 to 1). These results indicate that the gnotobiotic chicks stably maintained the phylogenetic diversity and predicted metabolic functionality of the inoculum community.

**IMPORTANCE** The domestic chicken is the cornerstone of animal agriculture worldwide, with a flock population exceeding 40 billion birds/year. It serves as an economically valuable source of protein globally. The microbiome of poultry has important effects on chicken growth, feed conversion, immune status, and pathogen resistance. The aim of our research was to develop a gnotobiotic chicken model appropriate for the study chicken gut microbiota function. Our experimental model shows that young germfree chicks are able to colonize diverse sets of gut bacteria. Therefore, besides the use of this model to study mechanisms of gut microbiota interactions in the chicken gut, it could be also used for applied aspects such as determining the safety and efficacy of new probiotic strains derived from chicken gut microbiota.

## Abstract (precis)

The domestic chicken is the cornerstone of animal agriculture worldwide, with a flock population exceeding 40 billion birds/year. It serves as an economically valuable source of protein globally. The microbiome of poultry has important effects on chicken growth, feed conversion, immune status, and pathogen resistance. The aim of our research was to develop a gnotobiotic chicken model appropriate for the study chicken gut microbiota function. Our experimental model shows that young germfree chicks are able to colonize diverse sets of gut bacteria. Therefore, besides the use of this model to study mechanisms of gut microbiota interactions in the chicken gut, it could be also used for applied aspects such as determining the safety and efficacy of new probiotic strains derived from chicken gut microbiota.

## INTRODUCTION

The chicken gut microbiota influences nutrient utilization (1, 2), immune development (3), endocrine activity (4), development of gastrointestinal tract (5), and detoxification, thus contributing to the improved performance of the birds. The chicken gastrointestinal tract harbors complex communities of bacteria (6, 7). The highest species diversity in the chicken gastrointestinal tract is observed in the cecum, which contains up to 10^11^/g organisms (8–10) and therefore has been widely studied. In addition to commensal bacteria, cecum also could harbor enteric pathogens that pose both avian and zoonotic health risks (11). The commensals could prevent the colonization of pathogens by competitive exclusion (12) and through the production of bacteriocins (13, 14).

Several experiments were conducted previously to study the microbial dynamics in broiler chicken intestinal tract (7, 15–17). Furthermore, studies were performed to determine the effect of gut microbes on feed utilization and conversion (1, 18). However, to determine the microbiome community composition, these experiments used 16S rRNA amplicon-based sequencing or culture-based techniques. 16S rRNA amplicon sequencing is inherently limited due to bias introduced during PCRs. Also, the data have lower resolution and are less efficient in predicting the functional properties of the microbiome. The accuracy of culture-based enumeration of the bacterial population is negatively affected by the inability to grow all the bacteria under culture conditions. In this respect, shotgun metagenomics provides a comprehensive representation of both taxonomical and functional properties of the microbiome. The two studies that used shotgun metagenomics for analyzing chicken microbiome were limited by the number of birds used in those experiments (6, 19).

Feral chickens are derived from domestic chickens that are released to the wild and that survive many generations. Living in the wilderness induces differences in the feeding habits and social behavioral patterns. Previous research in wild fowls and turkeys showed that the microbial communities in these birds differ considerably from those in the domesticated counterparts (20–23). Therefore, we hypothesized that the feral-adult microbiome could be substantially diverse from the microbial population of the commercial poultry. Use of feral-chicken microbiome as probiotic in commercial poultry practices could increase the diversity of the microbial populations and thereby possibly provide colonization resistance against enteric pathogens.

The objective of this study was to analyze the gut microbial colonization dynamics in gnotobiotic chicken (Gallus gallus) model under conditions of inoculation with the microbiome of adult feral chickens using shotgun metagenomics. Our findings suggested that the feral-chicken microbiome could colonize successfully in the young-chicken gut without causing detrimental health effects on the host.

## RESULTS

### Determination of *Salmonella* absence in the feral-chicken microbiota inoculum and germfree status of the hatchlings.

Gut samples were collected from the viscera of six feral chickens following slaughter. These samples were transferred to an anaerobic chamber, and the pooled cecum and colon contents of 6 feral chickens and the absence of Salmonella enterica in the samples were determined following the protocol described in the Bacteriological Analytical Manual (BAM), United States Food and Drug Administration. Briefly, samples were subjected to enrichment in tetrathionate broth followed by plating in xylose lysine deoxycholate (XLD) for agar plates. Absence of black colonies after 24 h of incubation at 37°C for 24 h was considered to represent a *Salmonella*-negative result. The sterility of the isolator and that of the hatchlings were determined by culturing fecal droppings and drag swabs from gnotobiotic isolators. All samples were found to be negative for bacterial colonies, indicating the germfree status of the chicks.

### Phylogenetic distribution of microbiome in gnotobiotic chicken gut compared to inoculum.

**(i) Microbiome composition in the inoculum and cecal contents at phylum level.** We sequenced each sample using paired-end 250-base or 300-base chemistry and an Illumina sequencing platform. The sequences generated per sample were on average 3.31 ± 0.43 Gb (mean ± standard error of the mean [SEM]). The details of the statistical data are provided in Table S1. A taxonomical abundance table based on reads after host read removal and quality filtering with phylum-level distribution was generated in MG-RAST server version 4.0.3 using the RefSeq database.

10.1128/mSphere.00035-19.1 TABLE S1 The overall statistics of data generation for each chicken sample in the current study. Download Table S1, XLSX file, 0.01 MB. Copyright © 2019 Thomas et al. 2019 Thomas et al. This content is distributed under the terms of the Creative Commons Attribution 4.0 International license.

**FIG 1:**
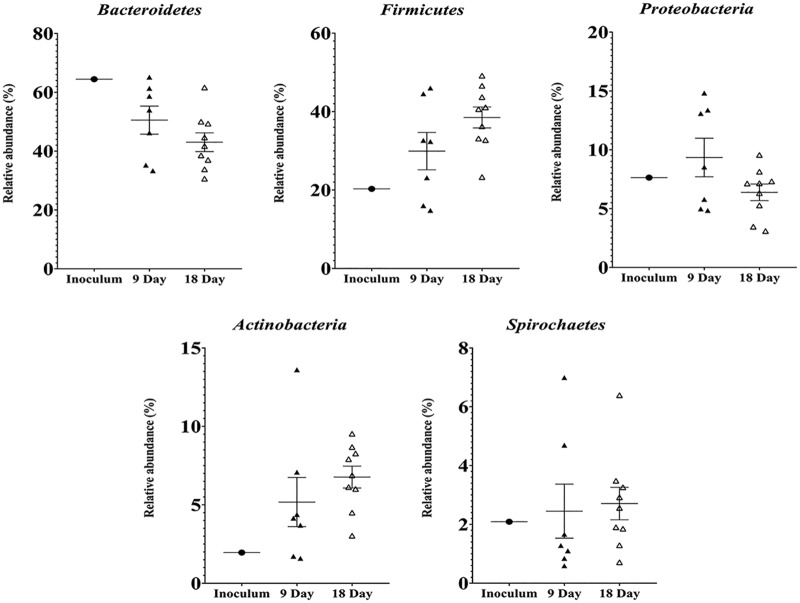
Taxonomical distribution of the major phyla in the inoculum and gnotobiotic chicken gut at day 9 and day 18. The inoculum was derived from 6 healthy feral chickens. Germfree chicks were inoculated on day 3 posthatch and euthanized on day 9 (*n* = 7) and day 18 (*n* = 9) posthatch. The five most abundant phyla were *Bacteroidetes*, *Firmicutes*, *Actinobacteria*, *Proteobacteria*, and *Spirochaetes*.

The five major phyla in all the samples were *Bacteroidetes*, *Firmicutes*, *Proteobacteria*, *Actinobacteria*, and *Spirochaetes* (Fig. 1; see also Table S2). The proportion of *Bacteroidetes* in the feral-chicken inoculum was 66.44% but was lower in the samples from day 9 (50.54% ± 4.77%) and day 18 (43.03% ± 3.19%). There was no significant difference between the day 9 and day 18 samples with respect to the levels of *Bacteroidetes* (*P* = 0.22). The abundance of *Firmicutes* in the inoculum was 20.3% and increased to 29.92% ± 4.77% in the day 9 samples and to 38.51% ± 2.67% in the day 18 samples. The *Firmicutes* abundance in the chicken gut did not increase significantly by day 18 compared to day 9 (*P* = 0.15). In addition, *Proteobacteria* levels increased at day 9 (9.34% ± 1.64%) compared to inoculum (7.63%) but decreased by day 18 (6.38% ± 0.7%) (*P* = 0.13), suggesting the role of facultative anaerobes in initial gut colonization (24). Similarly to that of *Firmicutes*, the abundance of *Actinobacteria* also increased temporally. In the inoculum, the fraction of *Actinobacteria* was 1.96%, whereas, in the day 9 and day 18 samples, the abundance increased to 5.18% ± 1.57% and 6.77% ± 0.7%, respectively (*P* = 0.38). The percentages of *Spirochetes* remained similar in the inoculum and gnotobiotic chicken samples at day 9 and day 18 (2.09%, 2.45% ± 0.92%, and 2.71% ± 0.55%).

10.1128/mSphere.00035-19.2 TABLE S2 Taxonomically assigned reads at phylum level in different samples. Download Table S2, XLSX file, 0.01 MB. Copyright © 2019 Thomas et al. 2019 Thomas et al. This content is distributed under the terms of the Creative Commons Attribution 4.0 International license.

**FIG 2:**
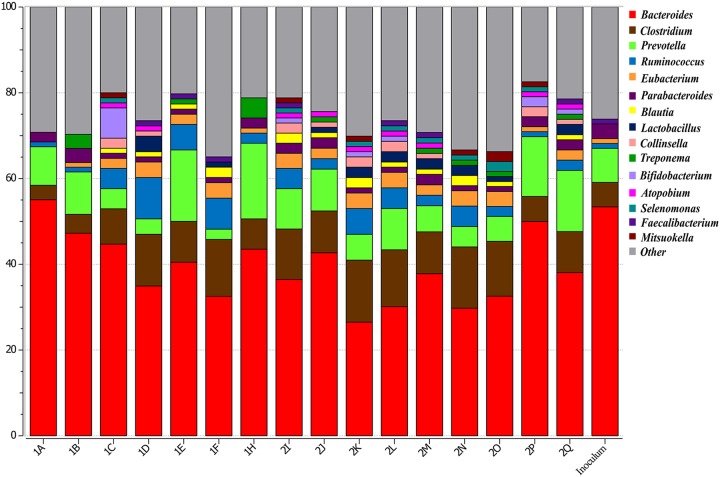
Genus-level distribution of gut microbiome in the gnotobiotic chicken inoculated with intestinal material from feral chickens. The pooled inoculum, derived from 6 healthy feral chickens, was orally inoculated to gnotobiotic chicken on day 3 after hatch. Birds were euthanized on day 9 (*n* = 7) and day 18 (*n* = 9) of age, and cecal contents were collected for DNA isolation. The metagenomic functional analysis was performed in MG-RAST using the RefSeq database with a maximum E value at 10^−5^ and minimum identity of 60%. Phylogenetic tables were generated in MG-RAST, and analysis was conducted using Explicet software.

**(ii) Microbiome composition in the inoculum and cecal contents at genus level.** At the genus level, the inoculum and the day 9 and day 18 samples were composed predominantly of *Bacteroides* (Fig. 2). However, the abundance was higher in the inoculum (47.78%) than in the day 9 samples (36.98% ± 2.87%) and day 18 samples (30.6% ± 2.12%). *Clostridium* levels increased in the day 9 (7.49% ± 1.15%) and day 18 (9.96% ± 0.76%) cecal contents compared to the inoculum (5.23%). The next-most-abundant genus was *Prevotella*, with the inoculum and gnotobiotic chicken samples showing similar percentages. *Eubacterium* levels increased from and abundance of 1.3% in inoculum to 2.4% ± 0.45% in day 9 samples and 2.99% ± 0.23% in day 18 samples. The abundance of *Ruminococcus* was higher in the day 9 samples (4.24% ± 1.11%) than in the inoculum (1.61%) and then decreased by day 18 (3.33% ± 0.4%). The level of *Parabacteroides*, which represented 3.68% in the inoculum, also decreased, similarly to *Bacteroides*, to 2.06% ± 0.29% and 1.98% ± 0.14% in the day 9 samples and the day 18 samples, respectively. The other three major genera whose levels increased in the cecal contents of gnotobiotic chicken compared to inoculum were *Lactobacillus*, *Collinsella*, and *Blautia* (Table S3).

10.1128/mSphere.00035-19.3 TABLE S3 The percentage abundance of different genera within the inoculum and day 9 and day 18 samples. Download Table S3, XLSX file, 0.01 MB. Copyright © 2019 Thomas et al. 2019 Thomas et al. This content is distributed under the terms of the Creative Commons Attribution 4.0 International license.

**FIG 3:**
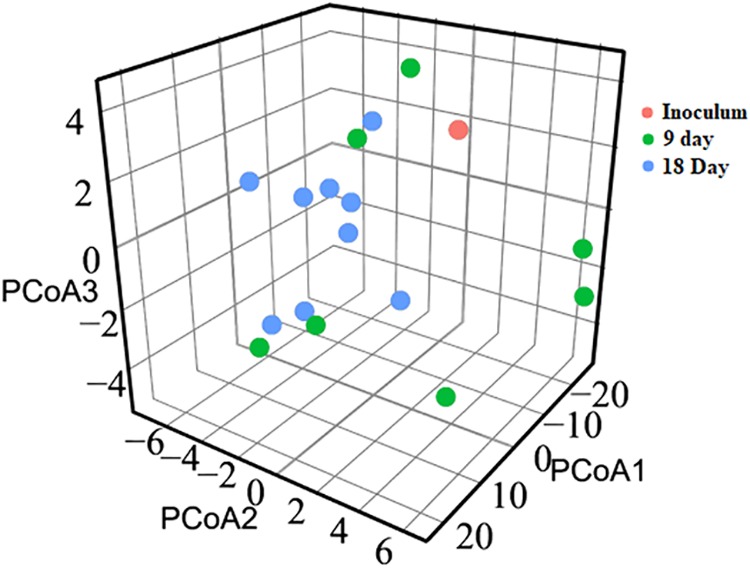
Principal-coordinate analysis (PCoA) of taxonomical diversity at the genus level in gnotobiotic chickens. Donor material derived from 6 healthy feral chickens was orally inoculated into gnotobiotic chicken on day 3 after hatch. PCoA analysis showed that the day 18 samples from inoculated gnotobiotic chicken were more closely distributed than the day 9 samples.

**(iii) Principal-coordinate analysis (PCoA) and β-diversity.** The principal-coordinate analysis (PCoA) data were calculated using Euclidean distance as the similarity metric for clustering the metagenomes (Fig. 3). While the day 9 communities were randomly distributed across space, the day 18 communities formed comparatively close clusters, which indicated that the microbial communities had evolved and matured temporally and had attained similar community profiles. These findings are similar to those revealing the microbial shift occurring in a previously uninhabited environment, such as infant gut, where the microbial community attains maturity and stability in the initial years of life (25).

**FIG 4:**
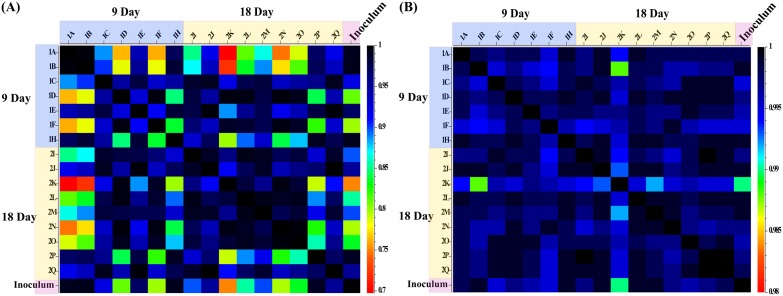
Comparison of taxonomical and functional β-diversities between feral-chicken-derived inoculum and gnotobiotic chicken gut samples on day 9 and day 18. The β-diversities were measured using the Morisita-Horn similarity index in Explicet software. The indices ranged between 0 and 1, where 1 is considered to represent similarity and 0 is considered to represent dissimilarity. Taxonomically, individual variations were observed between the inoculum and gnotobiotic chicken samples whereas the functional characteristics of the gnotobiotic chicken communities were closely similar to those of the inoculum.

Shotgun metagenomics was used to study the dynamics of the microbial community structure in the cecum of gnotobiotic chicken and the inoculum. The β-diversity represents the diversity between the samples with respect to the compositional units. The Morisita-Horn index values ranged from 0 to 1, where 1 indicates similar communities and 0 indicates dissimilar communities, and are given in Fig. 4A. All the values ranged between 0.7 and 1.0, indicating that the communities were more similar than dissimilar. However, individual variations in the colonization pattern were evident. For example, the communities in the samples from from birds 1C, 1D, and 1F on day 9 and birds 2K, 2L, 2M, 2N, and 2O on day 18 were dissimilar from the inoculum communities. However, the similarity between the samples with respect to the functional characteristics of the communities was higher (Fig. 4B). The Morisita-Horn index values ranged between 0.98 and 1, suggesting that the functional properties of the inoculum and the cecal samples collected at day 9 and day 18 from gnotobiotic birds were similar.

**(iv) Functional analysis of the cecal microbiome in the gnotobiotic chicken.** Analysis of functional categorization of the bacterial metagenome provides an understanding of the metabolic profile of the community. The metagenomic reads for feral-chicken inoculum and cecal samples of gnotobiotic chicken collected on day 9 and day 18 were assembled into contigs and then annotated against the MG-RAST server at different levels of hierarchy (Table S4).

10.1128/mSphere.00035-19.4 TABLE S4 Overall functional annotation of the samples at the subsystem level using mgRAST server version 4.0.3. Download Table S4, XLSX file, 0.4 MB. Copyright © 2019 Thomas et al. 2019 Thomas et al. This content is distributed under the terms of the Creative Commons Attribution 4.0 International license.

**FIG 5:**
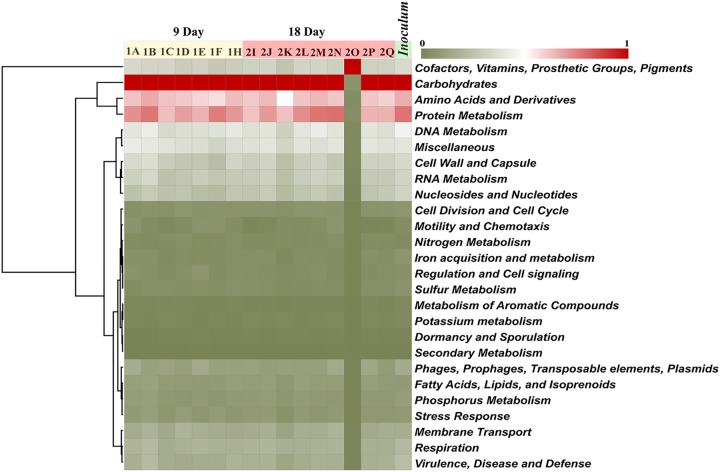
Predicted functional profile at the subsystem level of the microbiome in feral and gnotobiotic chickens. The pooled inoculum was derived from 6 healthy feral chickens. Birds were inoculated on day 3 after hatch and were euthanized at 9 days (*n* = 7) and 18 days (*n* = 9) of age, and cecal contents were collected for DNA isolation. The functional analysis was performed using subsystems information based on contigs from the MG-RAST database with an E value at 10^−5^, minimum identity at 60%, and a minimum read length of 100. Heat map was constructed in the Morpheus server (https://software.broadinstitute.org/morpheus) with a Euclidean distance matrix and average clustering method.

The overall distribution patterns analyzed at the subsystem level showed similar patterns for inoculum and gnotobiotic chicken microbiome collected on both day 9 and day 18 except for sample 2O due to the higher abundance of reads associated with genus *Brachyspira* (Fig. 5). The metagenome was enriched for enzymes involved in carbohydrate and protein metabolism. (Table S4). Other predominant functions belonged to categories such as DNA metabolism, RNA metabolism, cofactors, vitamins, and prosthetic groups (Fig. 5). These findings resembled the β-diversity determined for the functional characteristics, where all the communities exhibited similar profiles.

## DISCUSSION

The major objective of this experiment was to develop a gnotobiotic model to investigate the gut microbial colonization dynamics in the cecum of gnotobiotic chickens. Various methods of rearing gnotobiotic chicken have been described previously (17, 26–29). Gnotobiotic chickens have been reared using a custom-designed Gustafsson germfree apparatus (27). However, simpler methods were developed which made use of various disinfectants to reduce the bacterial load on eggs and sterile isolators (17, 26, 28, 29). Generally, the disinfectants used were mercuric chloride, quaternary ammonium, iodoform, and sodium hypochlorite solutions and commercially available chlorine dioxide solutions. In this study, Sporicidin was highly efficient in achieving disinfection without damaging the eggshells. Bacterial growth was not observed from samples collected from bird droppings and eggshells 2 days after hatching.

The conventionalization of gnotobiotic chickens using cecal microbial populations derived from adult chickens has been previously conducted (17, 30). The major shortcoming of those studies was that the microbial community was identified using culture-based technique and only a few organisms could be identified (17, 30). In this study, we used shotgun metagenomics to compare the microbiomes of the donor material derived from apparently healthy feral chickens and the gnotobiotic chickens. By enriching for the microbial genomic DNA, shotgun metagenomics could be successfully performed using a MiSeq Illumina platform (31). The findings from this study indicated that gnotobiotic chicken model, paired with next-generation sequencing techniques, could be an excellent tool to study the dynamics of gastrointestinal microbes in the chicken and could also be utilized in future experiments for studying the pathogenesis of enteric pathogens such as *Salmonella*.

The microbial population for inoculating gnotobiotic chickens in this study was collected from feral chickens that were *Salmonella* negative. The feral chickens originated from domesticated birds that had been released to the wild and had adapted to the wilderness through multiple generations. The process of feralization involves changes in social behavioral patterns, sexual selection, foraging requirements, and adaptation to predation in the wild. The expression of the genes that control these phenotypes also changes in the wild (32). Along with the host genetic changes, the microbiome could also diverge from that of the domesticated fowls. A study comparing the microbiome of wild and domesticated turkeys indicated that although the levels of diversity and richness of the microbial population were similar, only 30% of the operation taxonomic units (OTUs) were shared between them (23). In general, these results suggest that the gut microbiota composition of wild birds is more diverse and complex than that of the domesticated ones. This raises the possibility that introduction of these new species to the domesticated poultry could possibly alter the microbial community in a beneficial way in the fight against enteric pathogens. In this study, we found that the feral microbiome could successfully colonize in the young-chicken gut without causing problems such as reduced feed intake, diarrhea, or sepsis.

Metagenomics analysis of the cecal samples revealed that at the genus level, *Bacteroides* (47.78%) represented the most abundant organisms in the feral-chicken microbiome. The next-most-abundant organisms were *Clostridium* (5.23%) and *Prevotella* (7.12%), while *Ruminococcus* (1.61%) and *Lactobacillus* (0.34%) formed lower proportions. This contrasts with reports from studies of broiler chicken cecal microbiome where *Ruminococcus* and *Lactobacilli* were found to be the predominant genera. *Ruminococcus* species formed 15.6% of the total sequences in 3-day-old chicken cecum (16), and the proportion was 6% in 5-week-old chicken cecum (33). Similarly, *Lactobacillus* species were detected at 7% to 8% in broiler chicken cecum in those studies. A stable proportion of 16% to 23% *Ruminococcus* species in the total cecal microbiome, which did not alter with age, was observed by Ranjitkar et al. (7).

The gnotobiotic chicken microbiomes from this study showed that the proportions of *Bacteroidetes*, *Firmicutes*, and *Actinobacteria* in the samples collected at both day 9 and day 18 differed from the proportions in the microbiome of the feral-chicken inoculum. The abundance of *Bacteroidetes* was lower in conventionalized chicken than in the feral-chicken microbiome whereas that of both *Firmicutes* and *Actinobacteria* was higher. Furthermore, in the inoculated germfree chickens used in this study, the proportion of phylum *Bacteroidetes* was initially high and later decreased by day 18 with an increase in *Firmicutes*. Furthermore, the abundance of *Firmicutes* and *Actinobacteria* increased with the increasing age of chicks. The differences in feed and in the ages of the birds could possibly explain these variations in the colonization profile. Dietary intervention is a primary driving force that causes alterations in the microbiome (34, 35). Feral chickens forage in the wild on a variety of feed, including insects, berries, and worms, while the gnotobiotic chicken was fed on poultry starter diet. Another reason for the discrepancy between the feral and gnotobiotic chicken microbiome profiles could be that the inoculum was derived from pooled colon and cecal contents of feral chicken, while the analysis of gnotobiotic chicken microbiome was performed using solely the samples that were collected from the cecum.

Another finding was that although the proportion of *Proteobacteria* was higher at 9 days of age, it decreased with age and reached a level lower than that in the inoculum by day 18. This shift is analogous to the microbial dynamics in human infant gut, where initial colonization is by *Enterococcus* and *Escherichia* followed by *Bifidobacterium* and further by obligate anaerobes belonging to *Firmicutes* and *Bacteroidetes* (36–38). Similarly, a higher proportion of *Escherichia* was reported in young chicken which was later replaced by obligate anaerobes (7, 39). However, the initial colonization by *Proteobacteria* in broiler chicken can represent a public health risk, especially in the context of infection by enteric pathogens such as *Salmonella* and *Campylobacter*. An early bloom in such pathogenic *Proteobacteria* populations in broiler chicken may not be sufficiently countered by the late colonizers, thus resulting in the risk of infection even at market age (17). In this study, a decrease in the abundance of *Proteobacteria* was correlated with an increase in the population of *Firmicutes* and *Actinobacteria* on day 18. Our findings suggest that early administration of adult-feral-chicken microbiome could effectively prevent prolonged colonization of facultative anaerobes in chickens.

The microbial profile given in Fig. 2 shows the interindividual variation. The differences between individual birds were more pronounced at day 9 as indicated by the PCoA plot data (Fig. 3A). Similar variations in microbial composition between the experimental birds have been reported previously (1, 2). Similarly to our findings where the samples from 18-day-old chickens clustered much more closely, the microbial communities from older broiler chicken were previously reported to cluster with less variation than the communities from younger birds (39). In that experiment, the inoculum served as the sole source of microbes and successful colonization of the microbiome happened by 9 days of age.

In contrast, the functional properties of the microbial communities were more stable at day 9 and day 18 and were similar to those seen in the feral-chicken inoculum even while the microbial compositions were different. There were no significant differences between the inoculum and the day 9 and day 18 samples with respect to functional properties even at level 2 in the hierarchy determined using the SEED Subsystems database. Similar results for functional properties of chicken cecal microbiomes were observed previously (6, 19, 39).The variability between individuals for the taxonomic profile occurring during the microbial dynamics was not reflected in the functional profiles in these studies. It has been found that microbes occupying equivalent niches share similar functional properties even in diverse hosts (40). The microbial assemblage characteristics in a previously uninhabited habitat could be driven by equivalence in functional aspects rather than by the stochastic nature of microbial colonization. In this study, with the host niches being similar, the evenness in functional properties of the communities despite taxonomical variability could be explained only if functionally similar organisms were occupying equivalent niches.

Chickens act as a reservoir for enteric human pathogens, especially *Salmonella*. Recently, various *Salmonella* serotypes such as Enteritidis, I,[5],12:i:-; Typhimurium; Heidelberg; Hadar; Mbandaka; Montevideo; Agona; and Infantis have been found to be associated with *Salmonella* outbreaks (41, 42). Those rampant multistate *Salmonella* outbreaks due to transmission from live poultry reveal the necessity of pursuing studies aimed at control of *Salmonella* in poultry. The presence of enteric pathogens in poultry was controlled by using antibiotic feed additives (43). With the recent FDA regulations designed to limit the use of antimicrobials in the food supply due to public health concerns, use of such antibiotic feed additives is currently highly controlled. It is pertinent to develop alternatives such as prebiotics and probiotics that could manipulate the microbial community in chicken and thus competitively exclude enteric pathogens. The pioneering work by Nurmi and Rantala in 1973 demonstrated prevention of growth of *Salmonella* by competitive exclusion in the adult chicken microbiome whereas the microbiome from young birds was incapable of preventing the growth of *Salmonella* (17). The recent outbreaks suggest that this subject should get renewed attention as there is evidence indicating that more *Salmonella* serotypes have been adapting to chickens and causing a potential threat to public health (41). Since our results show that feral-chicken gut microbiota could colonize germfree chickens, our results raise the possibility that such a complex community might exclude the colonization of pathogens such as *Salmonella* in these birds. Our model could also be used for determining the mechanistic effect of microbiota subcomponents by conducting polyassociation studies by inoculating simple to complex defined gut bacterial species. The gnotobiotic chicken model developed here could also be used for determining the safety and efficacy of new probiotic species by conducting monoassociation or polyassociation studies.

## MATERIALS AND METHODS

### Experiment, sampling, and *Salmonella* detection.

Feral chickens were obtained locally near Brookings, SD, USA. The feral flock was once a captive flock of mixed breed and has been feral for no less than 8 years. The birds forage on a small grain farm and in surrounding grasslands. Feral chickens were sampled during a routine slaughter for personal meat consumption by the land owners. Gut samples from six birds were collected from the viscera following slaughter. The intestine was ligated at distal ileum and distal colon, maintained in ice, and transported immediately to the laboratory. Protocols used in this study for sample collection were reviewed and approved by the Institutional Animal Care and Use Committee (IACUC) at South Dakota State University, Brookings, SD. For processing, one portion of the sample for sequencing was frozen at −80°C, the remainder of each sample was transferred to a Coy anaerobic chamber, and the contents were expelled into 50-ml sterile conical tubes. Samples were diluted 1:10 (wt/vol) using anaerobic brain heart infusion broth supplemented with volatile fatty acids and vitamins (BHI-M), mixed by repeated pipetting, and aliquoted into cryovials. Anaerobic dimethyl sulfoxide (DMSO) was added at 18% (final concentration) and stored at −80°C until inoculation into young chickens. We used the detection protocol described in the FDA Bacteriological Analytical Manual (https://www.fda.gov/food/foodscienceresearch/laboratorymethods/ucm070149.htm) for determining the absence of *Salmonella* in the feral-chicken cecal samples. Briefly, 5 g of fecal sample from each chicken was added to peptone water and incubated aerobically at 37°C overnight. We then transferred 1 ml peptone mixture to 24 ml selective enrichment broth with tetrathionate brilliant green broth (TTB) and incubated the reaction mixture at 37°C for 24 h. We then streaked these samples on xylose lysine tergitol-4 (XLT-4) agar plates and incubated the reaction mixture aerobically at 37°C for 24 h. Absence of black colonies in the plates after 24 h of incubation was considered to represent absence of *Salmonella*. For preparing the inoculant for inoculation into germfree chickens, *Salmonella*-free samples were thawed and samples of stock from 6 feral chickens were pooled at equal volumes and further diluted 1:10 using anaerobic phosphate-buffered saline (PBS).

Gnotobiotic chickens were reared using a modified protocol that was described previously (17). Eggs of White Leghorn chickens were acquired from a commercial hatchery, treated with Sporicidin disinfectant solution (Contec, Inc.) and sterile water, and incubated in an incubator (pretreated with Sporicidin) at 37°C and 55% humidity. Humidity was maintained using a 1% (wt/vol) aqueous solution of potassium permanganate. After 19 days of incubation, eggs were removed from the incubator and candled for viability confirmation. Viable eggs were transferred to a biosafety cabinet and dipped in Sporicidin solution for 15 s and then wiped with a sterile cloth saturated with sterile water. Eggs were then transferred to autoclaved egg trays, placed in sterile autoclave bags, and transferred immediately to the port of the isolator unit. Eggs were sprayed with 5% peracetic acid and, after 20 min of exposure, were transferred inside the isolators. Eggs were maintained at 37°C and 65% humidity until hatching occurred on day 21.

Following hatching, birds were provided sterilized water *ad libitum* and a gamma-irradiated starter diet (LabDiet 5065, Irradiated) designed to meet the nutrient requirements of young chickens (Table 1) and monitored daily. On day 3 posthatch, birds (*n* = 16) were inoculated orally with 300 µl of pooled cecal contents. Seven birds were euthanized using cervical dislocation on day 9, and nine birds were euthanized on day 18 posthatch. The cecal contents were collected for DNA isolation and stored at −20°C until use.

**TABLE 1:** Nutritional composition and energy content of the LabDiet 5065 irradiated diet

Parameter	%
Composition	
Protein	22.1
Fat (ether extract)	4.2
Fat (acid hydrolysis)	5.2
Fiber (maximum)	2.8
Nitrogen-free extract	55.6
Minerals	5.3

Energy source	
Protein	25.4
Fat (ether extract)	10.7
Carbohydrates	63.9
Total Energy (kcal/g)	3.48

To assess the sterility of the isolator, swabs were collected on day 2 posthatch from the egg shells, droppings, and isolator floor and transferred to anaerobic transport media (44) and removed from the isolator. The swabs were then streaked on BHI-M agar plates and incubated aerobically at 37°C. The plates were examined for the presence of bacterial colonies after 24 h and 48 h of incubation.

### Genomic DNA isolation from the cecal contents.

Genomic DNA was isolated using a Powersoil DNA isolation kit (Mo Bio Laboratories Inc., CA). Briefly, approximately 100 mg of cecal contents was transferred to bead tubes and samples were homogenized for 2 min using a TissueLyser (Qiagen, Germantown, MD). DNA isolation was performed according to the manufacturer’s protocol, and DNA was eluted in 50 µl nuclease-free water. The quality of genomic DNA samples was assessed using NanoDrop One (Thermo Fisher Scientific, Wilmington, DE), and the samples were quantified using a Qubit 3.0 Fluorometer (Invitrogen, Carlsbad, CA). Samples were stored at −20°C until use.

### Microbial DNA enrichment and shotgun metagenomics sequencing.

Selective enrichment of bacterial genomic DNA was performed using a NEBNext microbiome DNA enrichment kit (New England Biolabs, Inc., MA) following methods previously published by our group (31). Briefly, 0.5 µg of genomic DNA was treated with 80 µl of MBD2-Fc-bound magnetic beads in the presence of binding buffer and incubated at room temperature for 15 min with rotation. After incubation, beads were separated by keeping the tubes on a magnetic rack for 5 min. The supernatant containing microbial DNA was transferred to a fresh tube. The DNA was further purified using Agencourt AMPure XP beads (Beckman Coulter) and stored at −20^0^C.

For shotgun metagenome sequencing, the enriched genomic DNA from pooled feral samples, seven samples from day 9 and nine samples from day 18 posthatch gnotobiotic chickens, was used. The concentrations of genomic DNA samples were adjusted to 0.3 ng/µl. Samples were then processed using a Nextera XT DNA sample prep kit (Illumina Inc., San Diego, CA) according to the manufacturer’s protocol. Purified products with unique barcodes were normalized using the bead normalization protocol of the manufacturer, and equal volumes of normalized libraries were pooled and diluted in hybridization buffer. The diluted libraries were subjected to heat denaturation prior to loading into the sequencer. Illumina paired-end sequencing was performed on the MiSeq platform using 2-by-250 paired-end sequencing chemistry.

### Sequence data processing.

The raw data files were demultiplexed and converted to fastq files using Casava v.1.8.2 (Illumina, Inc., San Diego, CA, USA). Reads aligning to chicken genome were identified using Bowtie 2 v2.3.0 (45). The reads were mapped against the chicken genome using Bowtie 2, and trimming was performed using SolexaQA++ (_V_3.1.7.1) with a phred score of 20 (46). The raw reads were then analyzed against the MG-RAST version 4.0.3 pipeline (47). The quality control steps in MG-RAST included dereplication, ambiguous base filtering, and length filtering. The taxonomical abundance was analyzed using MG-RAST with the best-hit classification approach using the RefSeq database, and parameters were limited to a minimum E value of 10^−5^, minimum identity of 60%, a minimum abundance of 50, and a minimum alignment length of 30 amino acids. The functional abundance was analyzed using hierarchical classification in MG-RAST and the SEED Subsystems database, and parameters were limited to a minimum E value of 10^−5^, minimum identity of 60%, a minimum abundance of 50, and a minimum alignment length of 30 amino acids. The OTU abundance tables were downloaded from MG-RAST and were used for downstream statistical analysis.

For functional analysis, after host read removal and trimming, the reads were assembled using Spades V3.11.1 with the “—meta” flag (48). The contigs that were less than 500 bp in length were removed from this study. Thereafter, we annotated the remaining contigs in MG-RAST server version 4.0.3 and functional profiling was performed at different hierarchical levels (E value = 10^−5^, minimum percentage identity of 60%, and a minimum alignment length of 100 amino acids). The heat map was constructed in the Morpheus server (https://software.broadinstitute.org/morpheus) with a Euclidean distance matrix and average clustering method.

### Statistical analysis.

The beta diversity between the feral-chicken inoculum and the day 9 and day 18 samples was estimated using the Morisita-Horn index in Explicet software (49). The PCoA analysis of taxonomical diversity was performed using Calypso version 8.72 (50). The differences between the day 9 and day 18 samples with respect to the phylum-level distributions were calculated in GraphPad Prism 8.0.1 (GraphPad Software, San Diego, CA) using the nonparametric Mann-Whitney U test. Significant differences were recorded at *P* values of <0.05. Genus-level distribution tables were analyzed using Explicet software.

### Data availability.

The data sets used in the current study are available in the NCBI SRA database under accession number PRJNA415593.
